# Low fitness is associated with abdominal adiposity and low-grade inflammation independent of BMI

**DOI:** 10.1371/journal.pone.0190645

**Published:** 2018-01-17

**Authors:** Anne-Sophie Wedell-Neergaard, Louise Eriksen, Morten Grønbæk, Bente Klarlund Pedersen, Rikke Krogh-Madsen, Janne Tolstrup

**Affiliations:** 1 The Centre of Inflammation and Metabolism and the Centre for Physical Activity Research, Rigshospitalet, University of Copenhagen, Copenhagen, Denmark; 2 The National Institute of Public Health, University of Southern Denmark, Copenhagen, Denmark; Vanderbilt University, UNITED STATES

## Abstract

**Objective:**

Up to 30% of obese individuals are metabolically healthy. Metabolically healthy obese (MHO) individuals are characterized by having low abdominal adiposity, low inflammation level and low risk of developing metabolic comorbidity. In this study, we hypothesize that cardiorespiratory fitness (fitness) is a determinant factor for the MHO individuals and aim to investigate the associations between fitness, abdominal adiposity and low-grade inflammation within different BMI categories.

**Method:**

Data from 10,976 individuals from the general population, DANHES 2007–2008, on waist circumference, fitness and C-reactive protein (hsCRP) were analysed using multiple linear and median quantile regressions.

**Results:**

In men, an inverse association between fitness (+5 mL min^-1^ kg^-1^) and waist circumference (-1.45 cm; 95% CI: -1.55 to -1.35 cm; p<0.001), and an inverse association between fitness (+5 mL min^-1^ kg^-1^) and hsCRP (-0.22 mg/L; 95% CI: -0.255 to -0.185 mg/L; p<0.001) was found, all independent of BMI. Similarly in women, an inverse association between fitness (+5 mL min^-1^ kg^-1^) and waist circumference (-1.15 cm; 95% CI: -1.25 to -1.0 cm; p<0.001), and an inverse association between fitness (+5 mL min^-1^ kg^-1^) and hsCRP (-0.26 mg/L; 95% CI: -0.3 to -0.22 mg/L; p<0.001) was found, all independent of BMI. Additionally, significant positive associations between waist circumference and hsCRP were found for both men and women, independently of BMI.

**Conclusion:**

Fitness was found to be inversely associated with both abdominal adiposity and low-grade inflammation independent of BMI. These data suggest that, in spite of BMI, high fitness levels lead to a reduction in abdominal fat mass and low-grade inflammation.

## Introduction

The use of BMI alone in predicting metabolic health is controversial [1][2][3]. Studies have shown that individuals can be obese and metabolically healthy (high insulin sensitivity, low abdominal adiposity and low inflammation level) or of normal weight with an unhealthy metabolic profile [1][3][4]. In fact, the MHO phenotype represents up to 30% of obese individuals [1]. However, major studies on this topic fail to consider the possible impact of cardiorespiratory fitness (fitness) on metabolic health [2][5].

Abdominal adiposity as well as low fitness have shown to be associated with a list of the most comprehensive and severe diseases including cardiovascular diseases [6][7], type 2 diabetes [8][9], cancer [8][10][11][12], dementia [13][14], depression [15][16] and additionally an increased risk of all-cause mortality independently of BMI [17][18]. Thus, the consequences of abdominal adiposity and low fitness are overlapping. Abdominal adiposity reflects the amount of visceral fat mass [4], which is more inflamed than subcutaneous fat[19][20]. It has been proposed that it may not be the amount of total body fat but the amount of abdominal fat, that is triggering an increase in both chronic systemic inflammation [21] and risk of metabolic disease [3][4].

A direct link between physical inactivity and accumulation of visceral fat has been established in intervention studies. These studies show that inactive subjects decrease their fitness level and accumulate visceral fat, without a change in total fat mass [22][23][24][25][26]. Based on this knowledge, high levels of fitness could be associated with a reduction in visceral fat mass and low-grade inflammation, leading to a good metabolic health in spite of obesity, known as the MHO phenotype. We therefore hypothesised that fitness was inversely associated with abdominal adiposity and low-grade inflammation independent of BMI.

The aim of the present study was to investigate the association between fitness and abdominal adiposity independent of BMI, the association between abdominal adiposity and low-grade inflammation independent of BMI and the association between fitness and low-grade inflammation independent of BMI.

## Method

### Study population and design

This study was based on data of 10,976 individuals from The Danish National Health Examination Survey 2007–2008 (DANHES 2007–2008) [27], undertaken in 13 different Danish municipalities. DANHES 2007–2008 was conducted in accordance with the Helsinki Declaration and approved by the Ethical Committee for the Region of Copenhagen. Written informed consent was obtained from all participants.

The study was designed as a cross-sectional study focusing on physical activity among other lifestyle factors. Adults of 18 or older in these municipalities were invited by letter to complete two internet-based questionnaires. A random subsample of these individuals were invited to participate in a health examination (n = 180,103). The basic questionnaire was fully or partially completed by 76,484 and a total of 18,065 individuals (7,360 men and 10,705 women) participated in the health examination. During the health examination, height, body weight, and waist circumference were measured and blood samples were collected.

Based on an initial screening interview about health status and use of medication, the participants were invited to or precluded from a maximal cycle ergometer test, and maximal oxygen uptake (VO2max) was predicted from maximal power output in a progressive cycle ergometer test [28]. One or more of the following conditions excluded participation from the cycle test: Any heart related disease, chest pain or pressure, moderate hypertension (≥160/100mmHg), intake of anti-hypertensive medicine, any heart or pulmonary medicine, pregnancy, muscle, joint or skeletal complains. Measures of fitness was included in the analyses which differs from physical activity by measuring cardiorespiratory endurance in contrast to energy expenditure [29]. Both fitness and physical activity have been shown to be inversely associated with the clustering of metabolic abnormalities, however, fitness have been proposed to exceed physical activity in its ability to identify risk factors of CVD [30] which is why fitness was chosen for the present study.

**Fig 1:**
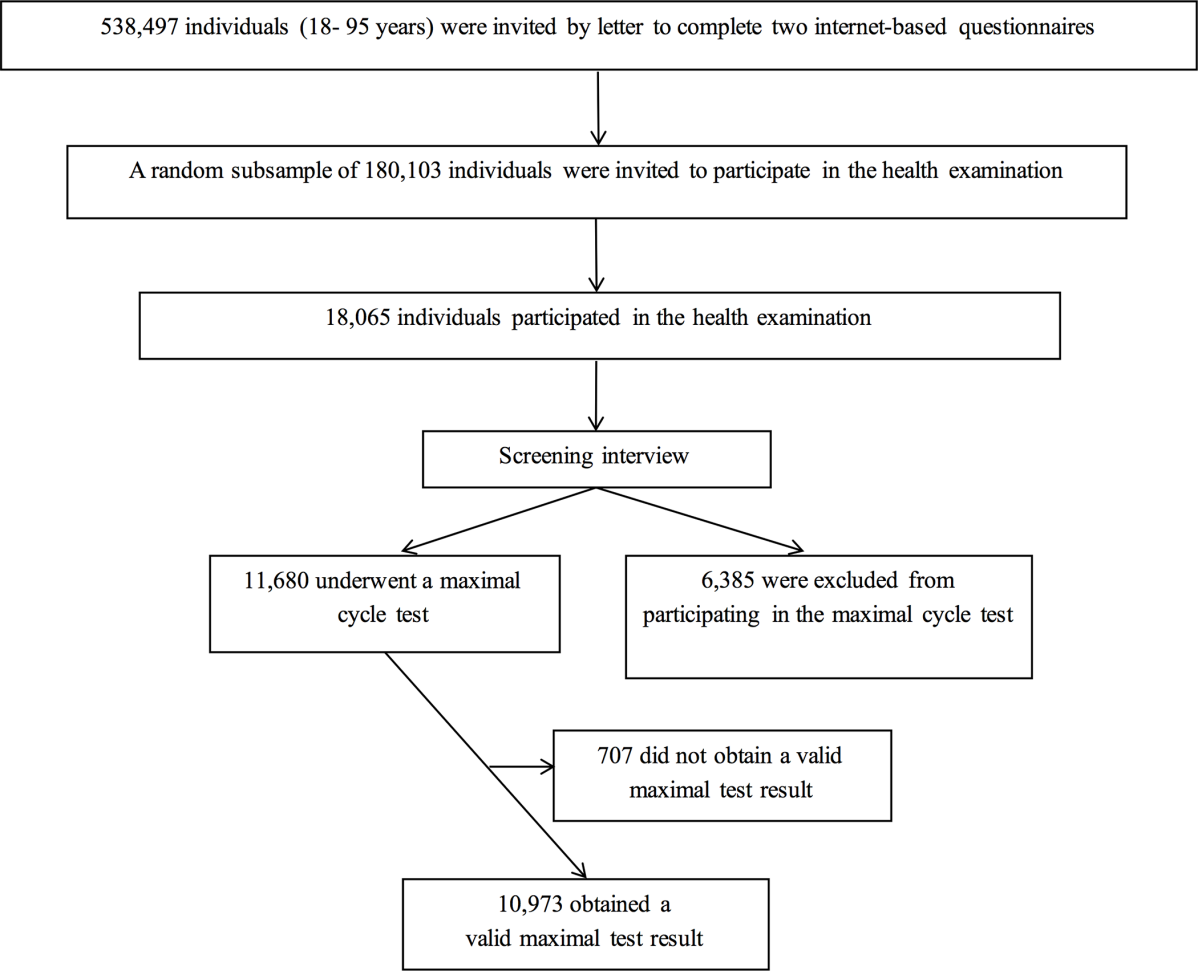
Selection of subjects.

A total of 11,680 participants (4,838 men and 6,842 women) underwent the maximal cycle ergometer test and 10,976 participants (4,638 men, 6,338 women) completed with a valid test result. Several criteria for reaching maximal exhaustion and completing a valid test was applied in the cycle test: the respiratory exchange ratio, levelling off (a plateau in oxygen uptake), and maximal heart rate [31]. Due to inconsistency in these criteria, a subjective evaluation of perceived exhaustion is recommended too [32]. To help participants reach maximal exhaustion, the test administrators verbally encouraged the participants to continue for as long as possible. Observation of the participant’s respiration, the Borg exhaustion scale [33], and the age-predicted maximal heart rate were used to decide whether maximal exhaustion was achieved. An invalid test result was registered if participants failed to reach their maximal aerobic capacity. Fitness was subsequently calculated from the estimated maximal oxygen uptake (VO_2_ max) divided by body weight [28] (Fig 1).

The 10,976 participants, who had a valid result from the maximal cycle ergometer test, were included in the analyses of this study. Measurements of high sensitivity C-reactive protein (hsCRP) led to errors for 160 men and 230 women, due to problems in drawing blood and errors in the analysis of blood samples.

### Waist circumference

Waist circumference was employed as an indirect measure of visceral fat as waist circumference has shown to be strongly associated with the amount of visceral fat (as measured by both magnetic resonance imaging and computed tomography) [4].

Waist circumferences were measured once with body tape (Chasmors WM02 Body Tape, Chasmors Ltd., London, United Kingdom). The participant was asked to take off their clothes, except for light underwear. The participant was asked to stand with their feet fairly close together and breathe normally; the reading of the measurement was taken at the end of exhalation. The waist was measured at a level midway between the lower rib margin and the iliac crest with the tape wrapped around the body in a horizontal alignment. Waist circumference was recorded to the nearest centimeter.

### C-reactive protein

Non-fasting venous blood samples were collected from fossa cubiti after light stasis in Lithium-heparin tubes. All tubes were stored at 5°C and transported weekly to the Department of Clinical Biochemistry, Rigshospitalet where the analyses were performed (Tosoh G7, Tokyo, Japan).

HsCRP qualifies as a nonspecific marker of low-grade inflammation [34]. With C-reactive protein being produced as part of the acute-phase response to tissue injury, infection or inflammation, an on-going pathological process is indicated by values higher than 10 mg/L [35]. Individuals with hsCRP values above this limit could influence the analyses leading to erroneous conclusions of the associations. Few participants showed hsCRP values above this limit (men: n = 97, women: n = 158) and when excluding these participants from the analyses the trend or significance of our results did not change. Based on this observation the participants with hsCRP values above 10 mg/L were not excluded.

### Statistics

Multiple linear regression models were performed to investigate the association between fitness and waist circumference adjusted for BMI (linearly), age (linearly and squared term), education, smoking, self-rated health and alcohol consumption in men and women. Interaction between BMI and fitness was added to the model. Furthermore, the assumptions of linearity, homogeneity of variance and normal distributed residuals were confirmed.

Median quantile regression analyses were conducted to assess the association between waist circumference and hsCRP adjusted for BMI (linearly and squared term) and age (linearly and squared term) education, smoking, self-rated health and alcohol consumption in men and women. Quantile regression to model median hsCRP was preferred over linear regression because of the skewness of hsCRP, which could not be optimally corrected using standard transformations. A quantile regression, does not assume underlying normality but is more robust to normal errors and outliers [36]. Interaction between BMI and waist circumference was added to the model.

Median quantile regression analyses were also used when investigating the association between fitness and hsCRP adjusted for BMI (linearly) and age (linearly and squared terms) education, smoking, self-rated health and alcohol consumption in men and women. Also in this model, an interaction between BMI and fitness was added.

To prevent from residual confounding, adjustment of continuous confounders (BMI and age) was added to the models in both linear and squared terms[37]. All statistical analyses were performed using STATA 13.0.

## Results

Descriptive characteristics of the study sample were stratified by sex and presented as medians and interquartile range or total number and percentage (Table 1).

**Table 1:** Descriptive characteristics of the 10,976 participants from DANHES 2007–2008. Median (25^th^ -75^th^ percentiles) unless stated otherwise.

	Men (n = 4,638)	Women (n = 6,338)
**Age (years)**	49 (40–59)	47 (38–57)
**Body mass index (kg/m**^**2**^**)**	25.2 (23.3–27.4)	23.4 (21.5–26.1)
**Number of overweight** **(BMI≥25 and <30 kg/m**^**2**^**), No. (%)**	2026 (43.7)	1649 (26)
**Number of obese** **(BMI≥30 kg/m**^**2**^**), No. (%)**	413 (8.9)	458 (7.2)
**Waist circumference (cm)**	94 (87.5–100.6)	82.9 (77–90)
**High sensitivity** **C-reactive protein (mg/L)**	0.9 (0.3–1.8)*	0.9 (0.3–2.0)*
**Cardiorespiratory** **fitness (mL/min/kg)**	35.9 (30.7–41.7)	31.5 (26.6–36.7)
**Alcohol consumption (>14 units/ week for men, >7 units/week for women), No. (%)**	1062 (22.9)	1813 (28.6)
**Educational level (<10y), No. (%)**	319 (6.9)	416 (6.6)
**Selfrated health (poor), No. (%)**	42 (0.9)	71 (1.1)
**Daily smoking, No. (%)**	571 (12.3)	591 (9.3)
**Diabetes, No. (%)**	53 (1.1)	57 (0.9)

* HsCRP was successfully measured in 4,478 of the 4,638 men and in 6,108 of the 6,338 women.

### Association between fitness and waist circumference

**Fig 2:**
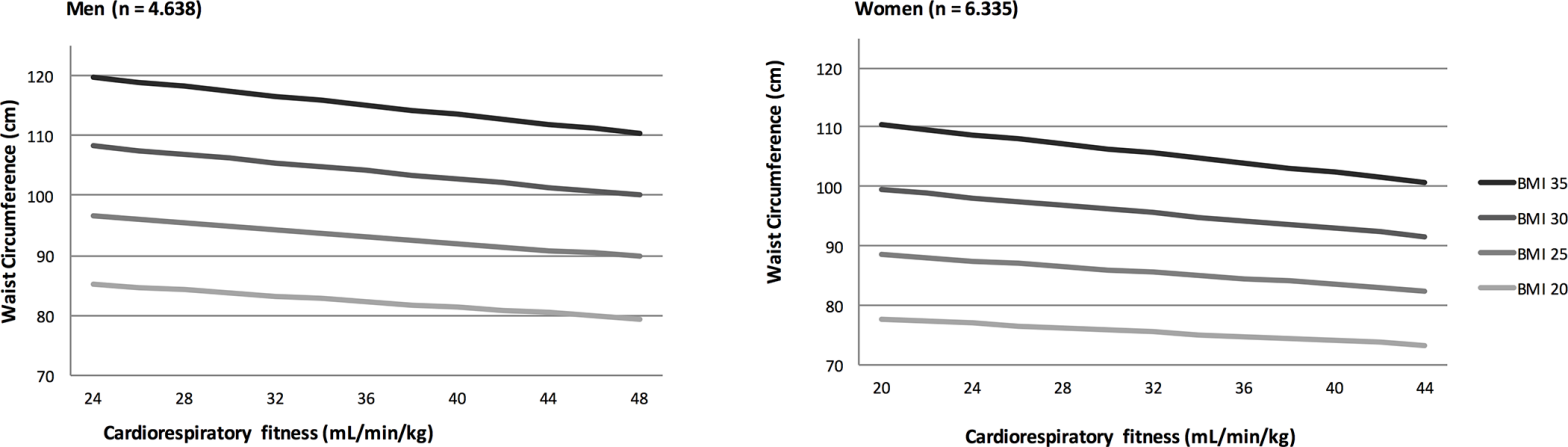
Waist circumference by cardiorespiratory fitness and BMI in men and women. Values are estimated, from the regression model, for a 40 year old woman or man with a BMI (kg/m^2^) of respectively 20, 25, 30 or 35.

In both men and women, higher levels of fitness were associated with lower waist circumference, independent of BMI. When BMI, age, education, smoking, self-rated health and alcohol consumption was kept constant, a higher fitness by +5 mL min^-1^ kg^-1^ was associated with a lower waist circumference by -1.50 cm (95% CI: -1,62 to -1,39 cm, p<0.001) in men and by -1.26 cm (95% CI: -1,39 to -1,13 cm, p<0.001) in women (Fig 2). Adjusted R-squared were 0.83 for men and 0.78 for women, meaning that all together fitness, BMI, age, education, smoking, self-rated health and alcohol consumption could explain 83% of the variance in waist circumference in men and 78% of the variance in waist circumference in women in this unselected population. An interaction between fitness and BMI was found for both men (-0.0098) and women (-0.0146) (p<0.001).

### Association between waist circumference and hsCRP

**Fig 3:**
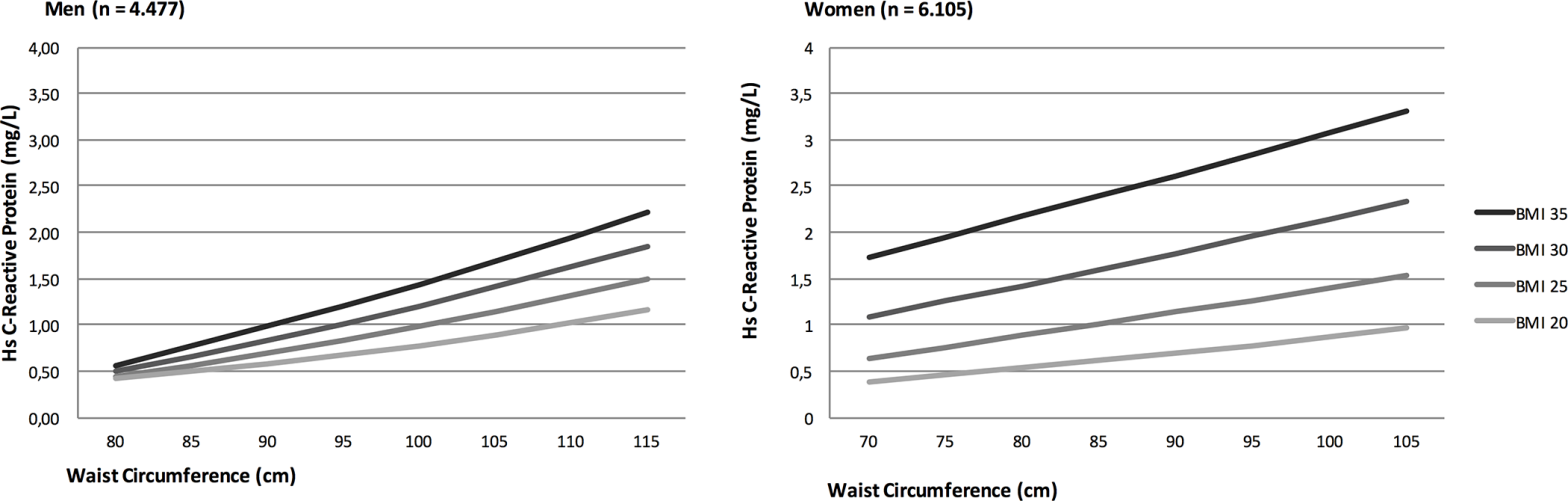
High-sensitivity C-reactive protein by waist circumference and BMI in men and women. Values are estimated, from the regression model, for a 40 years old woman or man with a BMI (kg/m^2^) of respectively 20, 25, 30 or 35.

Higher waist circumference was continuously associated with higher hsCRP, independent of BMI in both men and women. When BMI, age, education, smoking, self-rated health, and alcohol consumption were kept constant, a higher waist circumference by +1 cm was associated with a higher hsCRP by 0.03 mg/L (95% CI: 0.02 to 0.037 mg/L; p<0.001) in men and by 0.025 mg/L (95% CI: 0.017 to 0.034 mg/L; p<0.001) in women (Fig 3).

### Association between fitness and hsCRP

**Fig 4:**
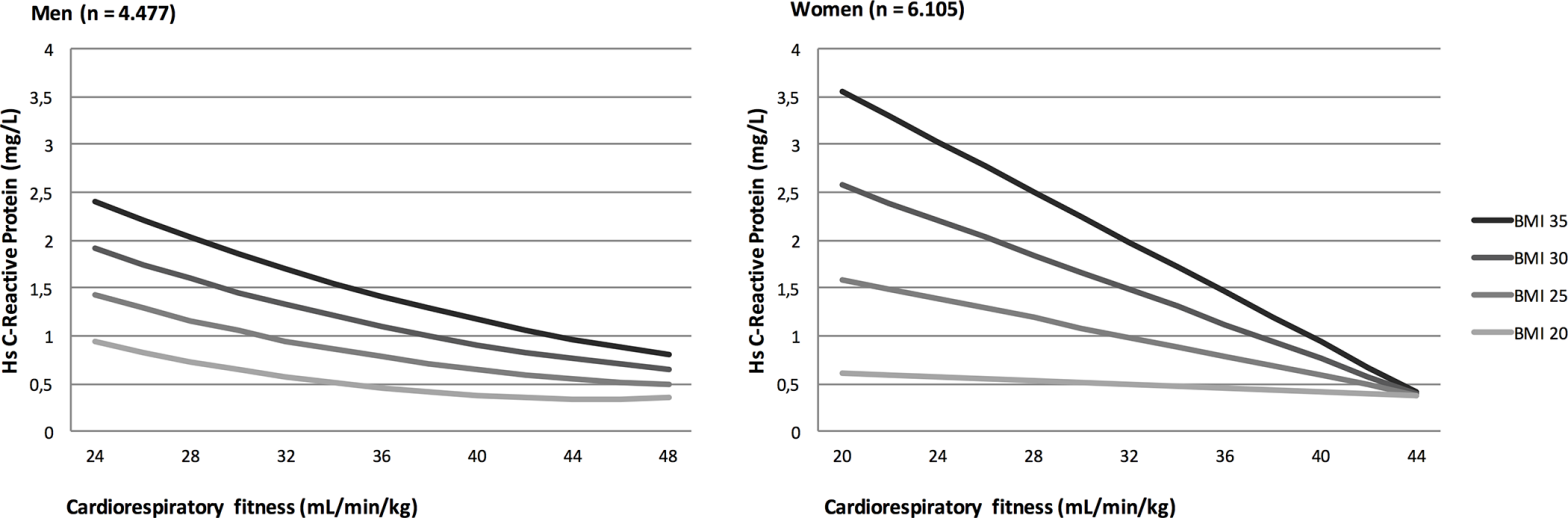
High sensitivity C-reactive protein by cardiorespiratory fitness and BMI in men and women. Values are estimated, from the regression model, for a 40 year old woman or man with a BMI (kg/m^2^) of respectively 20, 25, 30 or 35.

Higher levels of fitness were associated with lower hsCRP, independent of BMI in both men and women. When BMI, age, education, smoking, self-rated health, and alcohol consumption were kept constant, a higher fitness by +5 mL min^-1^ kg^-1^ was associated with a lower hsCRP by -0.19 mg/L (95% CI: -0.229 to -0.155 mg/L; p<0.001) in men and by -0.25 mg/L (95% CI: -0.29 to -0.21 mg/L; p<0.001) in women (Fig 4).

For both men and women an interaction between fitness and BMI was found (p<0.001), indicating that the association between hsCRP and fitness was stronger with higher levels of BMI (Fig 4).

## Discussion

In this cross-sectional study, we found a strong inverse association between fitness and waist circumference in both men and women adjusted for age, education, smoking self-rated health, alcohol consumption and most importantly BMI. Thus, the association was present in normal weight individuals as well as in overweight and obese individuals (Fig 2).

Furthermore, we found a positive association between waist circumference and hsCRP in both men and women in all BMI categories (Fig 3). In men, a low waist circumference was associated with a low level of low-grade inflammation independently of BMI. This association might reflect that men primarily accumulate fat located on the abdomen[38]. For women, low-grade inflammation seemed to be triggered by more than just abdominal adiposity. Despite a low waist circumference the women who had a high BMI, had a higher hsCRP. One explanation could be that other fat deposits, besides the abdominal fat, also give rise to low-grade inflammation, although to a lesser degree [39]. Women are to some extent protected against accumulating fat abdominally [4]. However, it could be that when women finally gain abdominal fat, low-grade inflammation reaches much higher levels, reflecting a higher risk of diseases compared to men [40].

Finally, we found an inverse association between hsCRP and fitness independent of BMI in both genders, showing that no matter BMI, if fitness levels were high then levels of low-grade inflammation were negligible (Fig 4). Although the association between fitness and hsCRP is statistically significant, the observed differences in hsCRP were small. However, even minor differences in hsCRP have shown to associate with risk of CVD and mortality [41][42], and our findings may therefore have clinical impact. Our results are in accordance with previous findings, showing that physical training reduces the level of systemic inflammation independent of weight loss [43], thereby improving the metabolic profile and decreasing the risk of disease. This BMI-independent anti-inflammatory effect of fitness may play a central role in promoting the metabolic healthy obese phenotype in both men and women.

In the present analysis, BMI was positively associated with the amount of abdominal fat and with the level of low-grade inflammation. Adjustments for BMI weakened the associations between fitness and abdominal fat as well as the association between fitness and low-grade inflammation, indicating that overall obesity contributes to both abdominal fat and low-grade inflammation. However, abdominal adiposity and low-grade inflammation were significant linked to low fitness, independent of BMI.

To our knowledge, no prior epidemiological study has investigated the BMI-independent association between fitness and abdominal fat, and related this to level of low-grade inflammation. Two cross-sectional studies [44][45] and a training intervention study [46] have previously examined the BMI-independent association between fitness and abdominal adiposity, however, the study populations were smaller and these studies were less informative since fitness was included as a binary variable. It was not possible to deduce the nuances of the association between waist circumference and fitness, and a link to low-grade inflammation was not included in these studies [44][45][46]. However, the studies [44][45][46] found an inverse association between fitness and waist circumference independent of BMI and are in accordance with the results of the present study.

When looking at the association between fitness and level of low-grade inflammation, the findings of this present study is consistent with, and even more prominent than findings in previous studies [47][48][49]. These previous studies included fewer participants (n< 1000), were limited by only including men [47], spanned a narrow age range (41-59y or 26y) [48][49], and included data from a submaximal measure of fitness [49].

A possible critique of the association between fitness and waist circumference could be that, when comparing individuals of different height but same BMI and VO_2_ max, the taller participants have physiologically larger waist circumferences and weigh more than their shorter counterparts. This results in the calculation of a lower fitness, since the formula for fitness is VO_2_ max divided by body weight. In contrast, less tall individuals have a smaller waist circumference, weigh less and thus, have a higher fitness. This could lead to a false correlation between fitness and waist circumference (Fig 2). However, the significant association between fitness and waist circumference persisted, when adjusting for height in the present study. A difference in body size is therefore not a plausible alternative explanation of our findings (regression models adjusted for body height are not shown). Another limitation of this study was the use of a cycle test to predict values of VO2max in the cohort. The cycle test was originally developed for health adolescence and young adults [28]. The burden of chronic conditions and physical limitations increase with age and contribute to a difficulty of measuring VO2max in older persons, however, due to our strict exclusion criteria, participants with muscle, joint or skeletal complains were excluded leaving only participants who were expected to be able to complete the test. Moreover, exercise testing by bicycle is considered to be an appropriate method to detect VO2max for elderly individuals as low fitness capacity and balance deficits does not hinder the test [31].

A total of 76,484 of the 538,497 adult individuals completed the questionnaire corresponding to 14%. Furthermore a number of 18,065 of 180,103 adults participated in a health examination, resulting in a participation rate of 10%. The low response rate could imply answers from a particularly fit segment of the population. Yet, given the sheer size of the study and when taking the average values from Table 1 into consideration, it is unlikely that the 18,065 participants should all belong to a particularly fit segment. Also, when looking at associated data, measures of fitness did show to be normally distributed.

Also by excluding "diseased" participants or those with comorbid conditions from the exercise test we artificially selected the test population and we need to take this into account as a limitation when comparing and transferring our findings into another cohort.

The present study identified a significant difference in the values for abdominal adiposity, fitness and low-grade inflammation, in people with the same BMI. However, to obtain a more accurate identification of individuals who are healthy despite obesity and individuals who are at metabolic risk despite normal weight, the presented data suggest that measurements of waist circumference, hsCRP, and fitness should be taken into account. Based on the overall results of the current study, we conclude that high fitness levels are inversely associated with the amount of visceral fat and the level of chronic systemic low-grade inflammation regardless of BMI.
